# Heterologous Production of 6-Deoxyerythronolide B in *Escherichia coli* through the Wood Werkman Cycle

**DOI:** 10.3390/metabo10060228

**Published:** 2020-06-01

**Authors:** R. Axayacatl Gonzalez-Garcia, Lars K. Nielsen, Esteban Marcellin

**Affiliations:** 1 Australian Institute for Bioengineering and Nanotechnology, The University of Queensland, Brisbane QLD 4072, Australia; r.gonzalezgarcia@uq.edu.au (R.A.G.-G.); lars.nielsen@uq.edu.au (L.K.N.); 2 The Novo Nordisk Foundation Center for Biosustainability, Technical University of Denmark, DK-2800 Kgs Lyngby, Denmark; 3 Queensland Node of Metabolomics Australia, The University of Queensland, Brisbane QLD 4072, Australia

**Keywords:** 6-deoxyerythronolide B, *Escherichia coli*, Wood-Werkman cycle

## Abstract

Polyketides are a remarkable class of natural products with diverse functional and structural diversity. The class includes many medicinally important molecules with antiviral, antimicrobial, antifungal and anticancer properties. Native bacterial, fungal and plant hosts are often difficult to cultivate and coax into producing the desired product. As a result, *Escherichia coli* has been used for the heterologous production of polyketides, with the production of 6-deoxyerythronolide B (6-dEB) being the first example. Current strategies for production in *E. coli* require feeding of exogenous propionate as a source for the precursors propionyl-CoA and *S*-methylmalonyl-CoA. Here, we show that heterologous polyketide production is possible from glucose as the sole carbon source. The heterologous expression of eight genes from the Wood-Werkman cycle found in Propionibacteria, in combination with expression of the 6-dEB synthases DEBS1, DEBS2 and DEBS3 resulted in 6-dEB formation from glucose as the sole carbon source. Our results show that the Wood-Werkman cycle provides the required propionyl-CoA and the extender unit *S*-methylmalonyl-CoA to produce up to 0.81 mg/L of 6-dEB in a chemically defined media.

## 1. Introduction

Natural products are our richest source of molecules with antimicrobial, antifungal, pesticide and anticancer activities [1]. They are usually associated with the secondary metabolism of organisms, these molecules are generally derived from coenzyme A (CoA), shikimate, mevalonate and 1-deoxyxylulose-5-phosphate intermediates [2]. Among the different classes of natural products, polyketides represent a large family of linear or cyclic poly-β-ketones produced by several microorganisms and plants [3]. They are synthesized by enzymatic complexes, known as polyketide synthases (PKS), through successive rounds of decarboxylative condensations between an acyl-CoA thioester and a β-carboxy thioester, in a manner that resembles fatty acid biosynthesis. Polyketides have a broad range of applications, from agriculture (as pesticides) to human health (as antibiotics, immunosuppressants and anticancer agents) [4]. PKS can be classified into three types. Type I or multimodular PKS (mPKS) work similarly to an assembly line, where each domain is responsible for the extension of the polyketide by the condensation and selective reduction of an acyl-CoA building block [5]. This particular feature makes them well suited for rational engineering. Type II polyketide synthases are aggregates of monofunctional proteins which condensing component resembles β-ketoacyl synthase II in bacteria. The last group, type III polyketide synthases are self-contained enzymes that form homodimers with an active site in each monomer that catalyzes the priming, extension and cyclisation reactions iteratively to form polyketide products. Type III PKS do not use acyl carrier protein (ACP) domains.

Polyketide production in native producers can be challenging. Native producers cannot always be cultured under laboratory conditions or fail to express the desired enzymes in bioreactors [3]. Many biosynthetic gene clusters are dormant under laboratory condition, reducing our ability to access the full potential of natural products discovery and manufacturing. To circumvent these issues, heterologous production of natural products is emerging as a viable alternative. However, many model organisms have a limited ability to supply precursors for heterologous and efficient production. The ability to synthesize precursors is particularly important for heterologous expression of silent biosynthetic gene clusters for which the final product is unknown.

**Figure 1:**
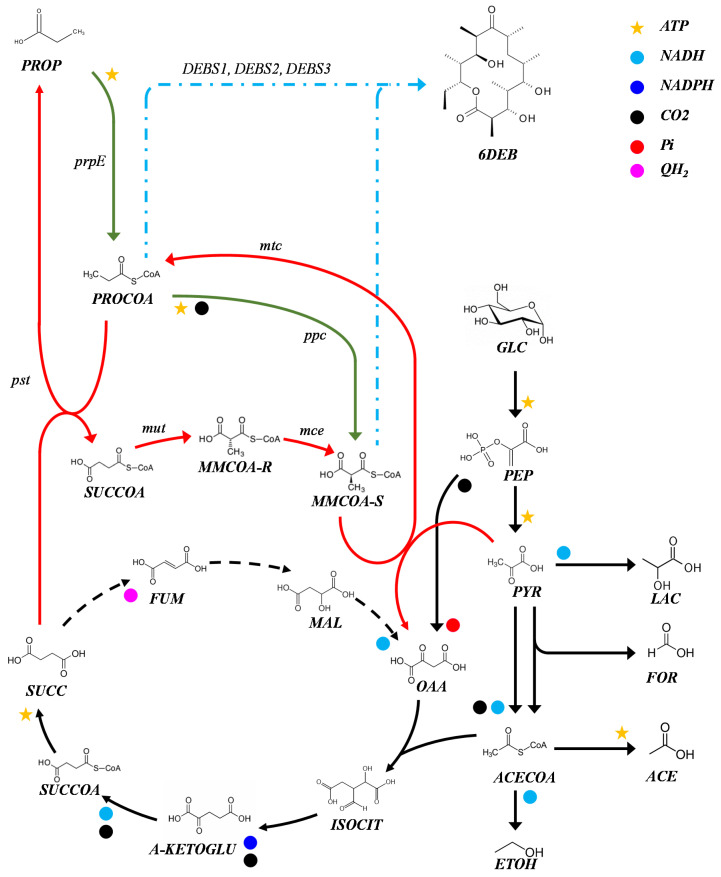
Production of 6-deoxyeritronolide B in *E. coli*. Key metabolites and cofactors are shown. *E. coli*’s native metabolism is shown in black. Engineered propionate assimilation pathway is shown in green. The Wood Werkman cycle is shown in red. 6-dEB synthesis is shown in blue. The main genes involved in the pathways sourcing precursors propionyl-CoA and *S*-methylmalonyl-CoA are shown. Under anaerobic conditions, the reactions shown in dashed lines occur in the reverse direction as indicated. Propionate assimilation occurs through a propionyl-CoA synthetase (*prpE*). Propionyl-CoA carboxylase (*ppc*). Mutase-epimerase pathway from *p. shermanii* (*mut, mce*). The wood Werkman cycle from *p. acidipropionici*: methylmalomnyl-CoA carboxyltransferase (*mtc*); propionyl-CoA:succinate transferase (*pst*); methylmalonyl-CoA mutase (*mut*); methylmalonyl-CoA epimerase (*mce*). GLC, glucose. PEP, phosphoenolpyruvate. PYR, pyruvate. LAC, lactate. ACECOA, acetyl-CoA. ETOH, ethanol. FOR, formate. ACE, acetate. OAA, oxaloacetate. ISOCIT, isocitrate. A-KETOGLU, α-ketoglutarate. MAL, malate. FUM, fumarate. SUCC, succinate. SUCCOA, succinyl-CoA. MMCOA-R, methylmalonyl-CoA (R). MMCOA-S, methylmalonyl-CoA (S). PROCOA, propionyl-CoA. PROP, propionate. 6DEB, 6-deoxyeritronolide B. Reactions are not balanced.

Several organisms have been proposed as hosts for the heterologous production of polyketides [6,7,8]. Among them, *Escherichia coli* has proven to be a robust cell factory for the production of polyketides like SEK26 from *Gibberella fujikuroi* [9] and the erythromycin precursor 6-deoxyerythronolide B (6-dEB) from *Saccharopolyspora erythraea* [10]. The latter being the first successful example of heterologous synthesis of polyketides. The synthesis of 6-dEB is performed by an mPKS called 6-deoxyerythronolide B synthase (DEBS) [11], which catalyzes the successive condensation of one molecule of propionyl-CoA and six molecules of *S*-methylmalonyl-CoA (Figure 1). While total synthesis of erythromycin C in *E. coli* has also been reported [4], the heterologous production of 6-dEB has received the most attention. This can be attributed to the fact that the performance of DEBS follows the same mechanism as most mPKS, using the same starter and extender units; therefore a better understanding and improvement of the heterologous production of 6-dEB may lead to a suitable platform for retrosynthesis of natural products [5].

Heterologous production of 6-dEB was initially achieved by introducing three DEBS genes into the engineered *E. coli* strain, BAP1 [10]. BAP1 can produce 6-dEB using external propionate as a source of propionyl-CoA and *S*-methylmalonyl-CoA through the activity of propionyl-CoA synthase (PRPE) and propionyl-CoA carboxylase (PCC) (Figure 1). Further metabolic engineering and high cell density fed-batch cultivation improved titers of 6-dEB production [12]. To date, the highest reported titer is 1.1 g/L, using an improved strain, *E. coli* K207-3 and plasmids pKOS207-129 and pBP130 [13] in a high cell density fed-batch reactor [14]. In all cases, propionate has been supplied to support polyketide production because *E. coli* is unable to produce the precursor *S*-methylmalonyl-CoA in the absence of propionyl-CoA carboxylase (PCC). Murli et al. [13] evaluated three different pathways to improve the availability of *S*-methylmalonyl-CoA—(1) propionate assimilation via propionyl-CoA synthase (PRPE) and propionyl-CoA carboxylase (PCC); (2) methylmalonyl-CoA synthetase pathway (*matB*); and (3) mutase-epimerase pathway from *Propionibacterium shermanii* (*mutA, mutB, mce*) (Figure 1) [13]. The PCC pathway supports the highest level of 6-dEB production (9.5 mg/L after 72 h) by directing the assimilated propionate towards the synthesis of the polyketide. Following that strategy, Vandova et al. evaluated 13 propionyl-CoA carboxylases from different species and showed that the PCC from *Streptomyces coelicolor* increased the final 6-dEB titer to 20 mg/L based on an input of 2.5 g/L of sodium propionate [15]. The relatively low yields from propionate suggest there is a limitation in the pool of propionyl-CoA and *S*-methylmalonyl-CoA that can be directed towards the polyketide synthesis. Moreover, the implementation of a two-substrate fermentation limits availability of the ATP required for the propionate assimilation and PKS assembly. In order to optimize the whole system, alternative pathways to source the acyl-CoA metabolites must be sought. Indeed, the citramalate and threonine pathways have been exploited for the production of erythromycin [16]; however, exogenous substrates are still required to sustain antibiotic production.

We have recently shown that *E. coli* can produce propionate via the Wood-Werkman cycle [17]. Through the cycle, pyruvate is carboxylated to oxaloacetate via a methylmalonyl-CoA carboxyltransferase (MTCA, MTCB, MTCC, MTCD) and then reduced to succinate through the reductive tricarboxylic acid cycle. Then, succinate is converted to succinyl-CoA by propionyl-CoA—succinate transferase (PST); this latter enzyme transfers CoA from propionyl-CoA, generating propionate as a by-product. Methylmalonyl CoA mutase (MUTA, MUTB) and epimerase (MCE) then convert succinyl-CoA to *S*-methylmalonyl-CoA, which is used by the carboxyltransferase to complete that the cycle (Figure 1). This pathway has the potential to provide the precursors for the production of 6-dEB, opening new strategies for the heterologous production of complex polyketides. Here, we demonstrate that the Wood-Werkman cycle can provide acyl-CoA precursors for the synthesis of polyketides. Our results show that the anaerobic pathway can sustain the production of propionate and 6-dEB from glucose.

## 2. Results

### 2.1. Assembling a Three-Plasmid System for the Production of 6-dEB in E. coli

We evaluated the feasibility of using a synthetic Wood-Werkman cycle as a source of propionyl-CoA and *S*-methylmalonyl-CoA, the intermediaries required for the synthesis of 6-dEB. Previously, we demonstrated that under anaerobic conditions, *E. coli* can produce propionate via the Wood-Werkman cycle using a plasmid system which encodes a single operon (10 kb) under the control of the P_BAD_ promoter [17]. We decided to use the same expression system in 6-dEB producer strain, *E. coli* 207-3. That strain was engineered to maximize the synthesis of the polyketide by removing the propionate degradation pathway encoded in the *prp* operon (∆*prpRBCD*) and introducing a propionyl-CoA carbolxylase (PPC) to direct the assimilated propionate towards methylmalonyl-CoA [13]. Production of 6-dEB in *E. coli* 207-3 requires the expression of DEBS1, DEBS2 and DEBS3 encoded in plasmids pBP130 and pKOS207-129, while propionate production requires the expression of the eight genes from the Wood-Werkman cycle encoded in plasmid pBAD_WWCV2. Since plasmids pBP130 and pBAD_WWCV2 share the same origin of replication, we cloned the 10 kb operon from plasmid pBAD_WWCV2 into plasmid pACYC138. This step was necessary to build a three-plasmid system with compatible origins of replication. We then generated strain K207-6-dEB_W (*E. coli* 207-3/pBAP130/pKOS207-129/pABAD_WWCV2). This new strain contains five heterologous genes controlled by the P_T7_ expression system and eight heterologous genes controlled by the P_BAD_ expression system.

### 2.2. Production of 6-dEB in E. coli with Propionate Supplementation

**Figure 2:**
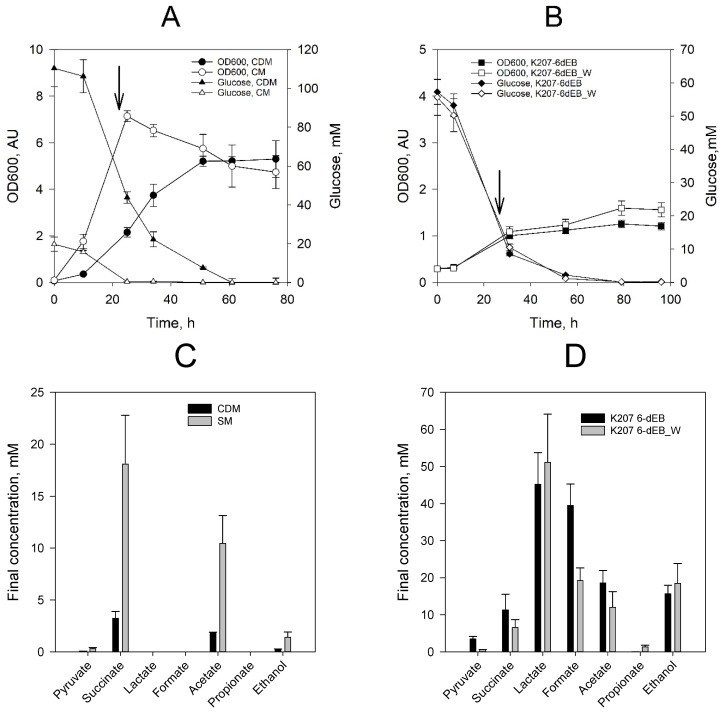
Batch cultures of *E. coli* K207-3 during the production of 6-dEB. (**A**) Aerobic fermentation performed in baffled flasks. The arrow indicates when the system was induced with IPTG (1 mM) and supplemented with propionate (10 mM). Complex SOC medium (SM) and chemically defined media (CDM). (**B**) Anaerobic fermentation performed in serum bottles in a chemically defined medium (CDM). The arrow indicates when the system was induced with IPTG (1 mM). All experiments were performed in duplicates. (**C**) Final fermentation products for aerobic cultures. (**D**) Final fermentation products for anaerobic cultures.

We used strain K207-6-dEB (*E. coli* 207-3/pBAP130/pKOS207-129) to produce 6-dEB as previously reported [13]. Production of 6-dEB in complex SOC medium (SM) and chemically defined medium (CDM)—Both with 10 mM propionate added—Were compared (Figure 2). Control fermentations were run at the same conditions using the backbone plasmids pBR322, pKOS207-129e and pACYC133 as shown in Table 1. Overall, control experiments grew faster than their respective counterparts did and none of them produced the polyketide. Growth profiles and final fermentation products profile appear in the Supplementary File. Cultures grew faster in SM medium and reached a higher OD600 during the first 24 h of growth at 37 °C (Figure 2A); growth then stopped and biomass concentration decreased after glucose depletion. Cultures grew slower in CDM medium, reaching stationary phase 50–60 h after inoculation. In both conditions, the production of 6-dEB was detected by LC-MS (Table 1). Samples for 6-dEB quantification were taken 24 h after the induction with Isopropyl β- d-1-thiogalactopyranoside (IPTG)and at the end of the fermentation. The 6-dEB spectra of the LC-TCI scan and the MS ion scan are included in the Supplementary File. The final 6-dEB concentration in SM medium was 6.24 mg/L compared to 2.68 mg/L produced in the CDM medium. No propionate was detected at the end of the culture period. Acetate was produced and re-assimilated. The final concentration of acetate in the cultures was below 3 mM in both conditions. Strain K207-6-dEB_W was not characterized in aerobic conditions because the activity of the methylmalonyl-CoA mutase (MUTA, MUTB) from *p. acidipropionici* is inhibited by oxygen.

### 2.3. Production of 6-dEB in E. coli without Propionate Supplementation

We tested 6-dEB production under anaerobic conditions in the strain expressing the synthetic Wood-Werkman cycle (K207-6-dEB_W) and compared it against the reference strain (K207-6-dEB) (Figure 2B). Strains K207-6-dEB and K207-6-dEB_W were grown anaerobically in CDM. L-arabinose was added at the beginning of the culture to express the Wood-Werkman cycle. At 24 h, the cultures were induced with IPTG and temperature was reduced from 30 °C to 20 °C. The temperature shift is required to improve the folding of the DEBS1, DEBS2 and DEBS3, that were induced by the addition of IPTG. Initial glucose concentration was reduced to 55 mM to avoid ethanol production. At this point, nearly 80% of the total glucose was consumed under both conditions. Propionate production was detected in the cultures harbouring pABAD_WWCV2 (1.11 ± 0.16 mM). The amount of 1-propanol was below the limit of detection. During the following 72 h of growth of both strains at 20 °C, glucose was depleted from the medium. Lactate and formate were the major fermentation products, followed by succinate, acetate and ethanol (Figure 1). The final propionate concentration in strain KOS207-6-dEB_W was 1.54 ± 0.23 mM. Liquid chromatography–mass spectrometry (LC-MS)analysis of the samples at the end of fermentation revealed that 6-dEB was produced only in the strains harboring plasmid pABAD_WWCV2, reaching up to 0.81 mg/L. The transition towards a glucose-fed anaerobic system has an impact on the final molar yield, as seen in Table 1. The yield achieved by culture K207 6-dEB_W represents almost a 45-fold decrease compared to the culture in aerobic conditions and SM medium (K207-6dEB). While the final titer is lower than the titer reached in SM medium, this investigation confirms the feasibility to produce 6-dEB from glucose without the addition of propionate and sets the path for future improvement.

## 3. Discussion

Current strategies for the heterologous production of 6-dEB in *E. coli* involve the use of high cell density cultures, the use of complex media and the addition of exogenous propionate in a two-stage process. The use of the Wood-Werkman cycle not only removes the use of exogenous propionate but also provide enough energy to sustain polyketide synthesis even at low cell densities, which implies higher specific productivity. This advantage comes with the limitation to operate in anaerobic conditions considering one of the enzymes within the cycle (mutase) is inhibited by oxygen, therefore limiting the availability of ATP required to support the synthesis of polyketides. However, the Wood-Werkman cycle provides an alternative for the generation of energy by allowing the operation of the electron transport chain by using fumarate as terminal electron acceptor [17]. Here, we tested whether that energetic surplus could make it possible to sustain polyketide production during a single-stage anaerobic process.

We initially evaluated aerobic conditions for the production of 6-dEB in complex and defined media with propionate supplementation. The K207-6dEB system produced around 6 mg/L (15 µM) of 6-dEB in complex medium, similar to a concentration reported previously [13]. Production decreased by more than 50% when the strain was grown in a CDM (Table 2). The higher yield in complex media is likely associated with an increased pool of propionyl-CoA and *S*-methylmalonyl-CoA from amino acid degradation, showing that heterologous PKS production in *E. coli* is likely limited by precursors availability [15]. In the K207-3 strain, 6-dEB precursors are supplied through the PRPE and PCC proteins from the external propionate; the complete consumption of 10 mM of propionate by the end of the fermentation indicates that it was predominantly converted into other, undesired products [13].

Propionate assimilation through propionyl-CoA synthetase (PRPE) consumes ATP, which makes the synthesis of 6-dEB an expensive process requiring 7 ATP per mole of the polyketide. Flux balance analysis indicates that the maximum molar yield of 0.11 mol 6-dEB per mol propionate is possible, as long as cellular maintenance is kept in balance with the oxygen uptake [18]. The same analysis using glucose as the sole carbon source was performed under aerobic and microaerobic conditions and using a linear pathway from succinyl-CoA to the precursors *S*-methylmalonyl-CoA and propionyl-CoA [18]. Although the overall yield was lower when using glucose, simulations reveal that the use of propionate can be avoided. Unfortunately, the glucose-dependent pathway requires additional activity through the pentose phosphate pathway and tricarboxylic acid cycle, which increases the maintenance requirements. This results in carbon loss in the form of CO_2_ which can be overcome by using the Wood-Werkman cycle under anaerobic conditions.

Our results confirmed that the joint expression of the synthetic Wood-Werkman cycle and the DEBS synthase genes in *E. coli* K207-3 supports the synthesis of 6-dEB from a sole carbon source (glucose), eliminating the need for exogenous propionate. The Wood Werkman cycle operates in anaerobic conditions, providing an additional electron sink via the reductive TCA cycle and maintaining a steady pool of the intermediaries R/S-methylmalonyl-CoA and propionyl-CoA. Introduction of the cycle into *E. coli* enabled anaerobic production of 6-dEB from glucose as a sole carbon source (Table 2). The titer obtained (0.81 mg/L) is still modest compared to the titers reached in highly engineered strains (up to 1.2 g/L) but it demonstrates that the use of exogenous propionate can be avoided. Our results are comparable with the recent reports where 6-dEB is produced in *E. coli* by engineering of the PCC pathway using different PPC complexes [15] and in engineered *Bacillus subtilis* strains [19]. Both reports still fed propionate into the system and reached yields of 2 mg/L (*B. subtilis*) and 20 mg/L (*E. coli*).

Efforts to engineer the native metabolism of *E. coli* to improve the availability of precursors for the synthesis of 6-dEB have previously been reported [16]. However, the results presented here show for the first time that polyketides can be produced from a sole carbon source in a recombinant host using an anaerobic pathway. It is unclear why a pathway such as the Wood-Werkman cycle has not previously been explored in a non-native host. We suggest this may be due to the low interest in the production of propionate compared to other higher-value compounds. However, while propionate production in *E. coli* is low, these levels are enough to source an additional pathway that leads to the formation of complex compounds, as we have shown here. Further engineering efforts should be made to improve the anaerobic metabolism of strain K207-3, coupling the activity in the cycle to the maintenance of cell requirements associated with the energy required for the assembly of the polyketide.

## 4. Materials and Methods

### 4.1. Strains and Plasmids

The full list of strains and plasmids used in this work are listed in Table 2.

### 4.2. Media Composition

Cells were routinely growth using LB medium (composition: 10 g/L yeast extract, 5 g/L bacto-tryptone, 5 g/L NaCl, pH 7.0; sterilized by autoclaving). Production of 6-dEB was performed using complex SOC medium and a modified M9 mineral medium.

SOC composition: 20 g/L bacto-tryptone, 5 g/L yeast extract, 0.6 g/L NaCl, 0.5 g/L KCl, 0.952 g/L MgCl_2_, 1.204 MgSO_4_, 3.6 g/L glucose. pH was adjusted to 7.0. Medium was sterilized by autoclaving. Glucose and MgSO_4_ were filter sterilized and supplied to the media after autoclaving.

Modified M9 mineral medium composition: 1.5 g/L KH_2_PO_4_, 4.34 g/L K_2_HPO_4_, 0.4 g/L (NH_4_)_2_SO_4_, 10 g/L glucose, 0.31 g/L MgSO_4_·7H2O, 1.5 mL/L vitamins solution, 1.5 mL/L salts solution. pH was adjusted to 7.0. Medium was sterilized by autoclaving. Glucose and MgSO_4_·7H2O were autoclaved separately. Vitamins and salts solutions were filter sterilized and then added to the medium. Salts solution composition: 27 g/L FeCl_3_·6H_2_O, 2 g/L ZnCl_2_·4H_2_O, 2 g/L CaCl_2_·6H_2_O, 2 g/L Na_2_MO_4_·2H_2_O, 1.9 g/L CuSO_4_·5H_2_O, 0.5 g/L H_3_BO_3_, 100 mL/L HCl. Vitamins solution composition: 0.42 g/L riboflavin, 5.4 g/L pantothenic acid, 6 g/L niacin, 1.4 g/L pyridoxine, 0.06 g/L biotin, 0.04 g/L folic acid, 10 mg/L thiamine, 0.27 mg/L cobalamin.

Antibiotics were supplemented to the medium when required using the following final concentrations—ampicillin, 100 μg/mL; streptomycin, 50 μg/mL; chloramphenicol, 30 μg/mL. IPTG (final concentration, 1 mM) and L-arabinose (final concentration, 10 mM) were also added to cultures when indicated. Yeast extract and bacto-tryptone were obtained from BD Diagnostics Systems (Franklin Lakes, NJ, USA). All other media components were obtained from Sigma-Aldrich Co. (St. Louis, MO, USA).

### 4.3. Plasmid Construction

Plasmid pABAD_WWCV2 was assembled using pACYC133 as a backbone. Plasmid pBAD_WWCV2 was digested with XbaI and HindIII. The 11-kb region 5’_PBAD-mutA-mutB-mce-mtcA-mtcB-mtcC-mtcD-pst_3’ was recovered from an agarose 1% gel using an extraction kit (QIAGEN, Hilden, Germany). The recovered fragment was cloned into the XbaI and HindIII sites in plasmid pACYC133. Ligation was performed using T4 DNA ligase (NEB) for 30 min at room temperature as indicated by the manufacturer. *E. coli* DH5α chemically competent cells (50 μL) (Bioline) were transformed with 5 μL of ligation mix by heat-shock at 42 °C. Cells were incubated at 37 °C in SOC media (1 mL) and plated on LB agar plates supplemented with chloramphenicol (30 µg/mL). Plates were incubated at 37 °C for 16 h. Plasmid pABAD_WWCV2 was recovered from a chloramphenicol resistant colony. Plasmid assembly was confirmed by restriction digestion and by Sanger sequencing (Australian Genome Research Facility, Brisbane, Australia). To build empty plasmid pKOS-129e, plasmid pKOS-129 was digested with XbaI and HindIII to remove DEBS1. The plasmid was recovered from gel and re-circularized.

### 4.4. 6-Deoxyerythronolide B Production in Aerobic Conditions

*E. coli* K207-3 harboring plasmids pBP130 and pKOS207-129 was streaked from a glycerol stock onto an LB agar plate plus ampicillin and streptomycin. The plate was incubated at 37 °C for 16 h. A colony was picked and grown overnight in LB media plus antibiotics at 37 °C and 200 rpm. One milliliter of the overnight culture was then used to inoculate 250 mL of LB media supplemented with antibiotics and grown at 37 °C for 24 h. At this point, IPTG was added to the medium and the culture was grown at 20 °C for 96 h. Media was supplemented with vitamin B12 (200 µM) and sodium propionate (10 mM). Cultures were performed in duplicates. Samples for organic acid quantification were taken 25 h after inoculation and at the end of the fermentation. 6-dEB was quantified as described in Section 4.6.

### 4.5. 6-Deoxyerythronolide B Identification and Quantitation

At the end of the fermentations, five mL of medium was centrifuged at 20,000× *g* for 5 min and the supernatant recovered. 6-dEB was extracted from the supernatant using the same volume of ethyl acetate. The organic fraction was recovered and dried using a speed vacuum. The residue was resuspended in 0.5 mL of methanol. 6-dEB was detected by LC-MS and quantified by LC-MS as previously reported [15]. Samples were diluted 1:50 in methanol. These diluted samples were analyzed by injection of 5 μL of the sample into the LC/MS system using a Zorbax RRHD Eclipse C18 column (50 mm × 2.1 mm × 1.8 μm). Mobile phases were 0.1% *v/v* acetic acid in either water (A) or acetonitrile (B). Elution was isocratic at 35% B for 2 min and increased to 100% B in a linear gradient from 2 min to 10 min. The column was subsequently washed with 100% B for 4 min and 35% B for 4 min. The mass spectrometer was an Agilent 6545 qTOF fitted with a dual AJS electrospray ion source in positive ion mode. 6-dEB eluted at 7.75 min.

### 4.6. 6-dEB Production from Glucose in Anaerobic Conditions

*E. coli* K207-3 harboring plasmids pBP130 and pKOS207-129 was transformed to harbor plasmid pABAD_WWCV2. A colony was picked and grown on SOC media supplemented with antibiotics (ampicillin, kanamycin and chloramphenicol). The overnight culture was used inoculate 160 mL of liquid media in a 170-mL serum bottle. Cells were grown in complex SOC medium and CDM medium supplemented with antibiotics and L-arabinose. Initial optical density at 600 nm (OD600) was adjusted to be ~0.3. Bottles were sealed and the headspace (10 mL) was flushed with N_2_ gas for 10 min to generate anaerobic conditions. Cultures were grown for 24 h at 30 °C and then cooled to 20 °C. IPTG was added to the media and cultures were grown for additional 72 h. OD600 was regularly monitored. Samples for organic acid quantification analysis were taken 25 h after inoculation and at the end of the fermentation. 6-dEB was quantified as described in Section 4.6.

### 4.7. HPLC Analysis of Organic Acids

Organic acids, carbohydrates and alcohol were quantified by ion-exclusion chromatography using an Agilent 1200 HPLC system (Agilent Technologies, Inc., California, USA) and an Agilent Hi-Plex H column (300 × 7.7 mm, PL1170-6830) with a guard column (SecurityGuard Carbo-H, Phenomenex PN: AJO-4490, Phenomenex, Torrance, CA, USA) as previously reported [17].

## 5. Conclusions

The optimal production of polyketides in non-native producers has long been studied with improvements in the production of the pool of acyl-CoA precursors being identified as a main bottleneck in production. Here, we showed that the Wood-Werkman cycle is a valuable strategy for 6-dEB production from a single carbon source. Further efforts to combine metabolic engineering and process control approaches are still required to improve performance.

## Figures and Tables

**Table 1:** 6-dEB production by recombinant *E. coli*.

Conditions	Relevant Genotype	Glucose ^1,2,^* (mM)	Propionate ^3^ (mM)	6-dEB ^4,^* (mg/L)	6-dEB Molar Yield ^5^
SM, aerobic	*E. coli* 207-3/pBR322/pKOS207-129e	26.9 ± 3.6	10	ND	-
CDM, aerobic	*E. coli* 207-3/pBR322/pKOS207-129e	105.9 ± 6.6	10	ND	-
CDM, anaerobic	*E. coli* 207-3/pBR322/pKOS207-129e	56.6 ± 2.89	-	ND	-
CDM, anaerobic	*E. coli* 207-3/pBR322/pKOS207-129e/pACYC133	53.9 ± 3.8	-	ND	-
SM, aerobic	*E. coli* 207-3/pBP130/pKOS207-129	19 ± 3.6	10	6.24 ± 1.74	1.71 × 10^−3^
CDM, aerobic	*E. coli* 207-3/pBP130/pKOS207-129	110 ± 9.6	10	2.68 ± 0.16	7.33 × 10^−4^
CDM, anaerobic	*E. coli* 207-3/pBP130/pKOS207-129	57.3 ± 3.7	-	ND	-
CDM, anaerobic	*E. coli* 207-3/pBP130/pKOS207-129/pABAD_WWCV2	55.7 ± 5.45	-	0.81 ± 0.08	3.98 × 10^−5^

^1^ Glucose consumed. ^2^ Complex SOC media contains 20 g/L bacto-tryptone, 5 g/L yeast extract and 3.6 g/L glucose, making it a very rich media and likely to reach high cell density cultures. Therefore, we selected a concentration of glucose that would provide similar final OD600. For anaerobic conditions, we halved glucose concentration to avoid carbon overflow. ^3^ Propionate supplied to media after 24 h. ^4^ 6-dEB concentration measured at the end of the fermentation. ^5^ Yield calculated considering the substrate that sources the acyl-CoA intermediaries ND not detected. * Results correspond to the average of two replicates.

**Table 2:** Bacterial strains and plasmids used.

Strain/Plasmid	Description	Reference
	Strains	
*E. coli* DH5α	Cloning host	Bioline (Aust) Pty Ltd., Alexandria, NSW, Australia
*E. coli* 207-3	K173-145 ygfG::T7prom-accA1-T7prom-pccB-T7term	[13]
	Plasmids	
pBP130	bla, T7prom-DEBS 2-ribosome binding site-DEBS3-T7term, ColE1 ori	[10]
pKOS-207_129	stp, T7prom-DEBS1-T7term, RSF1010 ori	[13]
pBAD_WWCV2	pBR322 derived plasmid. bla, araC-PBAD - mutA-mutB-mce- mtcA-mtcB-mtcC-mtcD-pst-tT7, ColE1 ori	[17]
pACYC133	cat, tet, p15A-ori	New England Biolabs, Ipswich, Massachusetts, MA, USA
pABAD_WWCV2	cat, tet, p15A-ori, araC-PBAD - mutA-mutB-mce- mtcA-mtcB-mtcC-mtcD-pst-tT7, p15A-ori	This work
pBR322	bla, ColE1 ori	New England Biolabs, Ipswich, Massachusetts, MA, USA
pKOS-207_129e	stp, RSF1010 ori	This work

## Supplementary Materials

The following are available online at https://www.mdpi.com/2218-1989/10/6/228/s1, Supplementary File 1: 6dEB.xlxs.
